# Great Expectations? Relation of Previous Experiences With Social Robots in Real Life or in the Media and Expectancies Based on Qualitative and Quantitative Assessment

**DOI:** 10.3389/fpsyg.2019.00939

**Published:** 2019-04-30

**Authors:** Aike C. Horstmann, Nicole C. Krämer

**Affiliations:** Social Psychology: Media and Communication, University of Duisburg-Essen, Duisburg, Germany

**Keywords:** human–robot interaction, social robots, expectations, uncertainty reduction, human–computer interaction

## Abstract

Social robots, which mostly look and behave like humans, are often perceived as somehow alive and treated similar to humans, despite the fact that they are non-living electronic devices. Based on considerations of the uncertainty reduction theory, the question arises what expectancies regarding social robots people have and what sources they use to achieve these expectancies. To receive an in-depth understanding of people’s expectancies regarding social robots and particularly how these expectancies are influenced by people’s experiences with real robots but also with fictional robots from media, thirteen semi-structured interviews and a quantitative online study (*n* = 433) were conducted. Results indicate that people’s experiences with robots in the media lead to high expectations regarding the skills of robots, which in turn increase people’s general expectancies regarding social robots being part of the society as well as their personal lives. Furthermore, knowledge of negatively perceived fictional robots increases negative expectancies of robots becoming a threat to humans, while technical affinity reduces general robot anxiety.

## Introduction

A social robot can be described as “an autonomous or semi-autonomous robot that interacts and communicates with humans by following the behavioral norms expected by the people with whom the robot is intended to interact” (Bartneck and Forlizzi, 2004, p. 592). In the future, this kind of robot will become increasingly omnipresent, which can be derived from the fact that the area of personal service robots has a high expected growth rate (Visti, 2018). Social robots are not necessary designed to be detailed replications of humans, for example due to the negative effects of the uncanny valley (Seyama and Nagayama, 2007). However, in accordance with their field of application, social robots are usually equipped with some humanlike features in their appearance and/or behavior to make the interactions with them more natural (Fong et al., 2003; for an overview of different types of social robots see Leite et al., 2013). Based on the media equation theory (Reeves and Nass, 1996), these behavioral and physical cues play a key role in people’s reactions toward media, which often rather resemble a reaction to a real person than to an electronic device. Against this background, the question arises which interhuman, social processes also apply to interactions with social robots.

One fundamental social process is described by the uncertainty reduction theory (Berger and Calabrese, 1975), which states that when interacting for the first time, people seek information about the other party to reduce their own uncertainty. People tend to process and organize these information by means of social categories, which are formed based on traits or social stereotypes (Andersen and Klatzky, 1987). These categories are associated with various kinds of further information, for example regarding typical behaviors, attitudes, and emotional reactions, and allow people to make predictions about others (Andersen and Klatzky, 1987).

Since social robots represent a new technology which is difficult to compare to previous technologies and thus hard to classify (Kahn et al., 2011), they probably evoke a lot of uncertainty. This is especially the case since social robots are electronic devices but also act and look like they are somewhat alive, which makes it particularly challenging to assign a clear category. Based on the assumptions of the uncertainty reduction theory (Berger and Calabrese, 1975), people probably also feel the need to predict and explain the behavior of social robots since these robots might adopt the role of an interaction partner in the future. Consequently, people likely attempt to build a general impression of social robots by considering all kinds of available information sources. It is important to explore and examine what kinds of expectations people have in regard to social robots, because these expectations have an influence on people’s perception and consequently their acceptance of the new technology. For example, people’s expectations regarding the usefulness as well as the ease of use of new technologies determines whether they will use these technologies or not (Davis, 1989; Venkatesh and Davis, 2000).

Despite this high relevance, very little is known about what people expect and, furthermore, often no distinction was made between what people actually expect and what they prefer. Thus, expectancy reports are usually mixed with preferences and were never deliberately contrasted before (e.g., Arras and Cerqui, 2005; Bruckenberger et al., 2013). Additionally, to understand people’s expectancies more thoroughly, mechanisms influencing the development of these expectancies should be considered. One pivotal question is, what sources of information people could possibly use to reduce their uncertainty and to form their expectancies regarding social robots. Here, science fiction is assumed to have a substantive influence since social robots are currently not very widespread and only few people come in contact with social robots (e.g., Bartneck, 2004; Bruckenberger et al., 2013; Sandoval et al., 2014). An additional question is, how people’s technical affinity and their locus of control when using technologies influence their formation of expectations.

In this study, qualitative interviews were conducted to find out more about people’s expectations of social robots with respect to their emotions as well as intentions and in contrast to their preferences. Furthermore, a quantitative online survey was planned to systematically examine possible influences on people’s expectancies. Specifically, their experiences with real robots, their reception of reports about real robots and their knowledge of fictional robots in science fiction movies or series were considered as potential information sources. Overall, a mixed method approach was chosen to achieve a more complete view by gaining depth as well as breadth on the subject.

### Uncertainty Reduction, Person Perception, and Social Categories

During the initial phases of an interaction between strangers, the interactors’ primary concern is to reduce the uncertainty which derives from not knowing the other person (Berger and Calabrese, 1975). Consequently, both sides aim to gather more information about the other side to predict and explain the other one’s behaviors (Berger and Calabrese, 1975). One way to reduce uncertainty and increase predictability of the other is the formation of a first impression of his or her personality (Andersen and Klatzky, 1987), which happens very fast and very easily (Asch, 1946). This process of impression formation is called person perception, which runs automatically and cannot be prevented (Asch, 1946). However, subsequent impressions can alter the first impression formed. Person perception often relies on social categories, which are used to predict potential behaviors, emotional reactions, personality attributes, attitudes, and values (Andersen and Klatzky, 1987). Social categories can be built upon trait concepts, whereby certain traits are assumed to have predictive power, or upon social stereotypes, where the affiliation with a certain social group is used to make assumptions (Andersen and Klatzky, 1987). Either way, both types of categories are used to easily and rapidly form an impression of the other and to reduce uncertainty at the same time.

Previous research has repeatedly shown that people tend to treat electronic devices such as computers (Reeves and Nass, 1996) and robots (Mumm and Mutlu, 2011; Eyssel and Hegel, 2012; Horstmann et al., 2018), but also virtual agents (Hoffmann et al., 2009) as if they were alive. Since social robots appear to be more than just machines but are still known as not being alive, it is challenging for people to assign a clear category to them (Severson and Carlson, 2010; Kahn et al., 2011). Consequently, making sense of the behavior of social robots and predicting how they will act as well as react to their environment poses a challenge. However, because of people’s aspiration to reduce uncertainty and to increase predictability, they will most likely attempt to form some kinds of expectations regarding social robots using whatever information sources are available to them.

### Perception of Social Robots

When looking at how people perceive and evaluate social robots, different concepts have to be considered. First of all, there is an extensive body of research on acceptance of social robots (e.g., Heerink et al., 2009; Eyssel et al., 2012; De Graaf and Allouch, 2013). Typical acceptance models from human–computer interaction are based on the three main aspects trust, security, and privacy; for human–robot interaction the social impact is added as a fourth aspect (Weiss et al., 2011a). Likewise, the factors for robot acceptance, which were identified by De Graaf and Allouch (2013), also have a focus on social and technical aspects and include usefulness, adaptability, enjoyment, sociability, companionship, and perceived behavioral control. In general, research has focused intensively on how variations of different characteristics of the robot (e.g., appearance: Goetz et al., 2003 and voice: Eyssel et al., 2012) have an impact on people’s acceptance.

Another focus of human–robot interaction are social reactions of users toward robots, which can be explained by the media equation theory (Reeves and Nass, 1996). Only a few cues are necessary to elicit automatic social reactions (Nass and Moon, 2000), for example speech, behavior, gestures, or appearance (Reeves and Nass, 1996; Powers and Kiesler, 2005; Powers et al., 2005), which are usually all present when interacting with a robot.

More important for the current research is the distinction between the two concepts preferences and expectancies, which were often mixed and never deliberately contrasted before. In general, a lot of research (Dautenhahn et al., 2005; Ray et al., 2008) has focused on people’s preferences regarding social robots, for example regarding their appearance (rather like machines and not like living beings), their communication modality (speech; in a human-like manner) and their traits (predictable, controllable, considerate, and polite). Altogether, people do not want robots to be too intelligent, but to more or less have the capacity to conduct limited actions according to their programs (Khan, 1998). Regarding expectancies, people generally expect robots to look and act appropriately according to the task context, thus their expectations depend on the context (e.g., playful for entertainment tasks, authoritative for serious tasks; Goetz et al., 2003). Furthermore, people expect lower levels of liking, higher levels of uncertainty, and less social presence when anticipating an interaction with a robot compared to a human (Spence et al., 2014). When people were asked to name characteristics of an imagined domestic robot, they rather mentioned performance-oriented traits (e.g., efficient, reliable, and precise) than socially oriented traits (e.g., feeling, compassionate, social; Ezer et al., 2009). Likewise, Arras and Cerqui (2005) surveyed over 2,000 people and found out that most people expect robots to be rather precise, reliable, rational, and perfectionist and rather not alive, human, and able to feel. In line with that, people think that social robots do not possess human-like personality or character traits (Dautenhahn et al., 2005). Regarding their functions, social robots are supposed to do simple, impersonal, non-creative, and repetitive household tasks (e.g., polishing windows, cleaning ceilings and walls, moving heavy things) rather than tasks involving some kind of human relationship (babysitting, watching pets, reading aloud; Khan, 1998; Ray et al., 2008). However, with all these results regarding characteristics, abilities, and tasks of social robots, it is not clear whether people actually stated what they expect or rather what they would prefer. Even though preferences appear to be projected onto expectations (c.f., Bartels, 1985) and may be derived from expectation-driven inferences (Wilson et al., 1989), they are still two different concepts, which should be looked at separately. For this reason, our aim was to systematically examine the difference between people’s preferences and expectancies regarding social robots by means of extensive interviews. Furthermore, since previous studies mainly focused on general characteristics, abilities, and tasks of social robots, for our interviews strong emphasis was placed on what role people expect social robots to play in their own personal daily lives and whether social robots are expected to have own emotions and intentions.

For the interviews, the following research questions were formulated:

*RQ1:* Where do people expect to encounter a social robot in their personal daily lives?
*RQ2:* Do people expect social robots to have own emotions and intentions?
*RQ3:* Are there differences between people’s expectancies and their preferences regarding social robots when these two are explicitly differentiated?

### Forming Expectations Regarding Social Robots

To understand how people form their expectations regarding social robots, it is important to look at where and how they have been in contact with robots. Some people interacted with a real robot before, for example due to their work field, at exhibitions, or as part of an experimental study. In general, however, not many people have yet interacted with a real social robot or a robot at all, mostly because they are currently not very common in today’s society (Van Oers and Wesselmann, 2016). As a consequence, a lot of people probably use other sources than their personal experiences to develop their expectations.

One source could be mass media since they are widely accessible. Also, they have proved to be an important means by which people, who had no direct experience with a certain technology yet, can receive information about this technology (Kriz et al., 2010; Sundar et al., 2016). Robots are often depicted in science fiction movies and series and these portrayals probably influence future human–robot relationships (Weiss et al., 2011b). According to Khan (1998), the general impression people have of robots appears to originate from science fiction, which is why it should help us understand humans in their relation to robots better. For instance, Asimov (1950), one of the most influential science fiction authors, presented the Three Laws of Robotics (minimizing harm to humans, self-preservation, and obeying orders), which address people’s most common fears when it comes to robots (McCauley, 2007). Furthermore, researchers have found that people often refer to science fiction movies and books when they are asked to discuss robots (Kriz et al., 2010). For instance, during several interviews conducted by Weiss et al. (2011b), people with no personal experiences with robots emphasized the influence of the mass media (e.g., movies) as well as advertisements on their attitude toward robots. Likewise, a survey by Bruckenberger et al. (2013) underlines the importance of mass media by showing that almost all participants knew robots from science fiction movies or from media coverage, which most of them believe to have shaped their general opinion about robots. In comparison, only some respondents stated to know real robots. Consequently, expectations of the capabilities of social robots in the real world could be biased by depictions of robots in the media (Sandoval et al., 2014), which may be fictional robots portrayed in movies or series, but also real robots appearing in the news, reports, or documentaries.

In line with that, people may expect the skills and abilities fictional robots exhibit also from real robots (Bruckenberger et al., 2013; Sandoval et al., 2014). The results of a study by Kriz et al. (2010) showed that in science fiction movies robots are reliably displayed with humanlike cognitive capabilities, which positively correlated with the respondents’ expectations regarding cognitive capabilities of real robots. However, real robots are far behind those fictional robots regarding physical and cognitive abilities (e.g., Murphy, 2018). Nevertheless, people probably derive some of their expectations about real robots’ skills and abilities from these fictional depictions, which in turn most likely influence their general expectations. However, the relationship between people’s different experiences with robots in real life or in the media and their expectancies regarding skills and the role of social robots has not been systematically examined before. Based on the indications previous research has given, we hypothesize that actual contact with real robots, the reception of reports or similar formats about real robots and the knowledge of fictional robots from science fiction movies or series will influence people’s expectations about skills and abilities of real robots and thus to what extent they expect interactions with social robots and social robots to be part of the society in the future.

H1: Previous contact with real robots, reception of media coverage on real robots, and knowledge of fictional robots in science fiction movies or series influence people’s expectations regarding (a) robot skills and abilities, (b) interactions with social robots, and (c) social robots being part of their daily lives.

### Fears of Social Robots

Science fiction movies usually promote either a “good” picture of human–robot interaction (e.g., robots as super-heroes trying to save the planet) or a “bad” picture (e.g., evil, intelligent robots trying to enslave mankind). This results in “weird”, double-minded feelings toward real robots (Bruckenberger et al., 2013). Khan (1998) listed as common topics of science fiction movies the dangerous machine, the want for life or consciousness, the public reaction to robots, and the overly intelligent robot. According to Khan, science fiction has a great influence on people’s image of social robots, which results in the two most common negative views on them: being constantly observed by robots and robots developing their own agenda including a revolt against humans. The fear that robots will become a competitor to humans and either replace or dominate them is also referred to as Frankenstein syndrome or complex (Asimov, 1947). In a study by Ray et al. (2008), people stated that they were worried about scenarios like autonomous robots, humans being replaced by robots, loss of control over as well as dysfunction of robots. All of these fears match typical science fiction scenarios. In Western cultures depictions of the dangerous and evil robot are more popular than for example in Japan, where robots may also be portrayed as good and fighting against evil humans or other evil robots (Bartneck, 2004). Thus, considering the previous findings as well as people’s Western cultural background, we hypothesize that people’s fears of social robots becoming dangerous for us humans will be influenced by their reception of fictional robots, especially negatively perceived ones, in science fiction movies or series. Since “good” and “bad” media representations of robots appear to impact the attitude of people in a different way (Bruckenberger et al., 2013) the aim here is to examine the differential influence of the negatively as well as of the positively perceived fictional robots. Additionally, people’s actual contact with real robots and their reception of reports or similar news coverage on real robots should also have an influence on those fears since here a more accurate picture of what real social robots are capable of should be obtained.

H2: People’s expectancies regarding the skills of robots as well as their previous contact with real robots, their reception of media coverage on real robots, and their reception of positively and negatively perceived fictional robots in science fiction movies or series influence their fears (a) about social robots becoming a threat to humanity and (b) about social robots in general.

Besides the general influence of people’s experiences with robots in real life or through media, other factors may also have an effect on their expectancies. Since robots are technical devices it is important to consider people’s attitude toward new technologies and how competent they feel using these technologies. The term technical affinity or technophilia is used to describe to what extent a person is attracted by technical devices (Karrer et al., 2009). People’s technical affinity resembles their positive attitude toward new technologies and thus should also result in a more positive evaluation of robots. People who tend to be rather excited about new technologies and look forward to working with them, will probably have higher expectations regarding robotic skills, abilities, and interaction opportunities and at the same time will have less fears regarding social robots.

Locus of control when using technologies on the other hand describes whether a person feels rather capable of reaching a goal using technology or rather helpless and overwhelmed when handling technological devices (Gaul et al., 2010). People’s locus of control is related to how competent people feel when handling technological devices (Beier, 1999), which is probably influenced by their actual technological competence and knowledge. Hence, people with a high locus of control when using technologies are potentially more realistic about what robots are able to do in the near future, which is why they should have lower expectations about skills, abilities and interaction opportunities, but also less fears regarding robots. Based on these theoretical considerations, we hypothesize that people’s technical affinity and their locus of control when using technology will influence their expectations and fears regarding social robots.

H3a: People’s expectations regarding social robots are enhanced by their technical affinity and reduced by their locus of control when using technology.
H3b: People’s fears regarding social robots are reduced by their technical affinity as well as their locus of control when using technology.

## Qualitative Interviews on People’s Expectancies Regarding Social Robots

### Methods

Thirteen individual in-depth interviews were conducted at a large European University. A semi-structured interview guideline was used and the interviews lasted between 23 and 58 min. The size of the sample was chosen based on the empirical findings that saturation for homogenous groups often occurs with about twelve participants (Guest et al., 2006). Participants were recruited in various Facebook groups. In total, seven female and six male students from different disciplines with an age range from 20 to 31 years (*M* = 24.62, *SD* = 3.53) attended the interview. To maintain general comparability, for example regarding experiences, only students with a similar European cultural background were interviewed. However, participants were also carefully selected to have various backgrounds, e.g., different study programs. The ethics committee of the division of Computer Science and Applied Cognitive Sciences at the Faculty of Engineering of the University of Duisburg-Essen approved the study and written informed consent was obtained.

After some warm-up questions about the general attitude toward new technologies and previous interactions with robots, a definition of social robots (robots who are able to interact socially with a human) was given. Since one aim was to separate expectancies from preferences, participants were specifically asked to describe what they expect and not what they would like to see happen in the first part of the interview and told that their preferences will be discussed in the second part. First, participants were asked to describe their typical daily routine and to explain during which of their everyday activities they expect to interact with a social robot in the future and during which they do not expect any interactions. Furthermore, the interviewees were asked more generally in which fields they expect to come across social robots and what kinds of robots they expect in about 50 years. In particular, participants were asked what kind of abilities and behaviors they expect and whether they expect social robots to have own emotions and intentions. Second, to talk about participants’ preferences, they were asked which abilities and behaviors they would like a social robot to be equipped with and whether it should have own emotions and intentions if they could decide freely. To find out more about how people specifically perceive a social robot with own intentions, respondents were asked how they feel and think about an imagined scenario where their personal robot suggests doing something it would prefer to do before doing an assigned task. The last question was about the most important advantage or benefit and the most important disadvantage or risk of social robots (see Supplementary Table S1 for the exact interview questions). At the end, aim and purpose of the study were explained to the participants and their time and effort were compensated (either with money or course credits).

### Results

After the interviews were transcribed, the respondents’ answers were grouped into categories using an inductive coding scheme and content-analytically analyzed employing the coding software MAXQDA. Intercoder reliability was calculated with about 25% of the data (3 out of 13 interviews) and two judges using the coefficient “Kappa”. The results indicate a substantial agreement (κ = 0.77, κ = 0.71, and κ = 0.63).

Half of the participants (*n* = 6) said that they were generally open toward new technologies, while the other half (*n* = 7) stated to be open but also skeptical. Most interviewees had contact with a non-social robot before, e.g., an industrial or vacuum cleaning robot (*n* = 7), others interacted with a social robot before (*n* = 3) and the rest did not encounter any kind of robot before (*n* = 3).

#### Expectancies Regarding Social Robots in People’s Personal Daily Lives

In regard to their personal daily lives (RQ1), many participants stated that they expect social robots to help with household chores (“*to have free time to get other important things done*”; *n* = 12), food preparation (*n* = 12), assistive work-related tasks (*n* = 7), and unpleasant or strenuous activities like carrying heavy things (“*to make people’s life easier or let’s say rather less stressful*”; *n* = 6). Several respondents mentioned that they expect to use social robots for company or entertainment (“*to repress loneliness*”; *n* = 6) and for assistance during recreational or sport activities (*n* = 5). A few said that they picture social robots helping them to fall asleep (e.g., by reading a story) or to wake up (*n* = 4) and with body care (*n* = 2). When asked what the participants expect social robots not to do, replacing social contacts (“*a robot cannot replace these interactions*”; “*people prefer real humans*”; *n* = 10), replacing the participants at their work or studies (*n* = 5), being present while the participants sleep or watch TV (*n* = 5) and while they engage in sport activities (*n* = 5) are listed.

#### Expectancies Regarding Own Emotions and Intentions of Social Robots

With regard to emotions (RQ2), many participants answered that they expect social robots to imitate human emotions (*n* = 8) and to recognize and react to those emotions (*n* = 7); only some reported to expect them to actually have own emotions (*n* = 3). Concerning own intentions (RQ2), a few respondents stated to expect social robots to have an own will and consciousness (*n* = 3) and to have only a limited decision-making scope (“*that it does not have the free choice but is guided by certain* aspects”; *n* = 3). However, when asked what participants expect social robots to not be able to, mostly having an own will or consciousness (“*they only have the will, we impose on them*”; *n* = 12), having own emotions (*n* = 10), and feeling empathy (“*to really know, whether another person is feeling bad and one should help him”; n* = 8) were mentioned. Also, some participants stated that they expect social robots to not be able to have negative feelings (*n* = 4) and feelings of love or attraction (“*extreme feelings, which make people do things, one doesn’t want*”; *n* = 4).

#### Comparison of Expectancies and Preferences

In general, most participants said they wish for a social robot which assists them in their daily lives (*n* = 10). When asked what they do not want, most respondents mentioned a social robot replacing or influencing their social contacts (*n* = 8). This basically reflects where most participants stated to expect interacting with social robots (household chores: *n* = 12; food preparation: *n* = 12) and not to interact with social robots in their daily lives (social activities: *n* = 10). Regarding robot abilities and characteristics, many participants explained that they would like social robots to be able to communicate with them (*n* = 8) and to move properly to fulfill their tasks (*n* = 4). Although many respondents said that they would like social robots to have some human-like features regarding appearance, voice, and communication skills (*n* = 7), many also said that they prefer social robots to be rather machine-like to differentiate them more easily from humans (“*because a machine is still a machine and not a feeling or human being*”; *n* = 8). This also goes in line with what is mentioned by the majority of the participants as expectations (communication skills: *n* = 11; motor skills: *n* = 9; human-likeness: *n* = 11; machine-likeness: *n* = 8). Most of the respondents reported to prefer social robots to neither have an own will or consciousness (“*it is hard to draw a line*”; *n* = 8) nor their own emotions (*n* = 8), which also basically corresponds with their expectancies (no own will: *n* = 12; no own emotions: *n* = 10). However, when comparing abilities which were only mentioned as expectations with abilities that were only mentioned as preferences, it can be noticed that people list more technological or machine-like abilities of social robots when talking about expectations (e.g., mathematic skills, environmental perception, knowledge database, and proactive thinking were only mentioned when asked about expectations) and more emotional and interpersonal abilities when they were talking about their preferences (e.g., empathy, positive emotions, helpfulness, anger/hatred, and fear/sadness were only mentioned when asked about preferences). Furthermore, about half of the interviewees stated that they would like a social robot to be able to have empathy and compassion (“*a person, who keeps you company and comforts you*”; *n* = 6), while, when asked about expectations, most of the respondents said that they expect social robots not to be able to be empathic (*n* = 8).

#### Further Results

As outlined before, one possible influence on people’s expectations regarding social robots could be science fiction themes, which is why we also assessed to what extent different science fiction robot movies and common science fiction scenarios were mentioned during the interviews. It was noticed that seven people mentioned fears of robots rising against humans, which is a common science fiction scenario, and nine actually referred to different robot movies during the interviews. Also, when asked about the most important disadvantage or risk of social robots, some respondents answered that humans may lose control over the robots, which could then revolt against the humans (“*that they develop an own will and start to think for themselves and maybe also rise against humans*”; *n* = 3).

### Summary of the Interview Results

In general, the interviewees were very open toward the idea of interacting with social robots in their daily lives and had many expectations but also preferences about where, when, and in what way these interactions may take place. Most of all, participants expect and prefer social robots to help them with household chores, food preparations or work-related tasks. However, most respondents did not imagine robots during social activities like meeting friends and family, which was also something they would prefer not to happen. Regarding skills and abilities, participants expect and would like social robots to be able to communicate and to move properly as well as to have human characteristics, but to be clearly identifiable as machines. Furthermore, social robots are expected as well as preferred to have no own emotions and no own will; nevertheless are they expected to imitate and react to emotions. The largest differences between expectancies and preferences was that people mentioned more technological abilities when asked what they expect and more emotional or interpersonal abilities when asked what they prefer. Moreover, the majority of respondents wished for a social robot to be empathetic and compassionate, although most do not expect a social robot to be able to be empathetic.

On top of these results, it was noted throughout the interviews that many respondents mentioned being afraid that social robots may develop an own consciousness and try to place themselves above humans, which represents a common science fiction scenario. Also, a majority of the interviewees mentioned various science fiction robot movies during the interviews. Since only a few interacted with an actual social robot before and no other sources of information were mentioned during the interviews, participants’ expectations seem to be greatly influenced by those science fiction movies, as other researchers also assumed (Khan, 1998; Ray et al., 2008; Weiss et al., 2011b; Bruckenberger et al., 2013). While the interview study helped to receive an in-depth understanding of people’s expectations and gave first hints regarding possible influences on these expectations, the aim of the online study is to examine these influences in a more systematical way. As the results of the interview study also suggest, the influences of experiences with real robots, depictions of real robots in media, and portrayals of fictional robots in science fiction movies and series are investigated in a broader manner by conducting a large-scale online study.

## Online Survey on Possible Influences of People’S Expectancies Regarding Social Robots

### Methods

An online study was conducted with a total of 451 participants, of which 18 had to be excluded due to being under age or unrealistically fast (less than 5 min) in completing the survey. The remaining sample consisted of 433 datasets, with 126 male (29.1%) and 307 female (70.9%) participants and an average age of 29.41 years (*SD* = 9.92), ranging from 18 to 69 years. The sample had a European cultural background and with regard to education, most participants possessed university entrance-level qualifications (37.4%) or a university degree (44.6%). The link to the online study was distributed publicly in various Facebook groups, on Internet platforms for online surveys and via personal messenger chats and E-Mail contacts. The ethics committee of the division of Computer Science and Applied Cognitive Sciences at the Faculty of Engineering of the University of Duisburg-Essen approved the study and written informed consent was obtained.

First, the aim and the context of the study were explained, then informed consent was obtained, followed by the questionnaires. At the end of the online survey, participants were given the opportunity to enter their email address to win an Amazon gift certificate before the study ended with a debriefing, where the purpose and aim of the study were disclosed. On average, participants needed about 15 minutes to fill out the questionnaire.

#### Contact With Real Robots

Participants were asked whether, how often (1 = “very rarely” to 5 = “very often”), and how intensively (1 = “briefly” to 5 = “intensively”) they had contact with industrial robots, domestic non-social robots (vacuum cleaner or lawn mower robots), social robots (autonomous robots which can interact and communicate with humans), or other robots before.

#### Reception of Reports About Real Robots

In a similar way, they were asked whether, how often, and how intensively they watched reports or similar formats about industrial, domestic non-social, social, or other robots before.

#### Reception of Science Fiction Movies or Series

First, people stated how often they watch movies or series where robots play an important role (1 = “very rarely” to 5 = “very often”) and how they would generally describe the relationship between humans and robots in those movies and series (5 items; e.g., “In science fiction movies/series robots are rather against humans.”; 1 = “strongly disagree” to 5 = “strongly agree”; α = 0.79). Then, they were asked which ones of the 16 robot movies or series presented in Table 1 they had seen and how well they remember them (1 = “barely” to 5 = “very good”). For all movies or series the participants had seen, they reported whether they remembered the main robot characters, also presented in Table 1, to what extent they perceive these robots as rather negative or positive (slider scale for an easier and more precise answer regarding the evaluation of the robots; 1 = “negative” to 101 = “positive”) and to what degree they expect future real robots to be like these robots (1 = “strongly disagree” to 5 = “strongly agree”).

**Table 1:** Robot science fiction movies and series and their main robot characters.

Robot science fiction movies/series	Fictional robot characters	Chosen based on
Terminator	T-800	1, 2, 3
	T-1000	
The Matrix	The Machines	1
Blade Runner	Roy Batty	1, 2, 3
	Rachael	
	Officer K	
2001: A Space Odyssey	HAL 9000	2
I, Robot	Sonny	1, 2, 3
	Other robots of the type NS-5	
Avengers: Age of Ultron	Ultron	4
Westworld	Dolores	4
	Maeve	
Star Trek: The Next Generation	Data	1, 2
Battlestar Galactica	The Cylons	4
Transformers	Bumblebee	1, 2, 3
	Megatron	
AI: Artificial Intelligence	David	1, 2
Wall-E	Wall-E	2, 3
Star Wars	R2-D2	1, 2, 3
	C-3PO	
	BB-8	
Ex Machina	Ava	4
Short Circuit	Number 5	1, 2
	The military robots S-A-I-N-T	
Forbidden Planet	Robby the Robot	2, 3

*1 = Kriz et al. (2010); 2 = Sandoval et al. (2014); 3 = (Sundar et al., 2016); 4 = IMDb search with the keywords robot, android and artificial intelligence (www.imdb.com)*.

#### General Expectancies

First, respondents stated how much they would like to interact with a robot (*contact intentions*; based on Eyssel et al., 2011; Eyssel and Kuchenbrandt, 2012; 5 items; e.g., “How much would you like to meet a robot?”; 1 = “not at all” to 5 = “very much”; α = 0.90). Based on the scenarios used by Eyssel et al. (2011) and the activities mentioned during the previously conducted interviews, 15 items to assess people’s expectations about future interactions with real robots were developed and used in the online study (*interaction expectations*; e.g., “I expect that I will be able to use a robot as fitness coach in the future.”; 1 = “strongly disagree” to 5 = “strongly agree”; α = 0.92). Also based on the statements of the interviewees and the assessment of mental capacities by Gray et al. (2007), people’s expectations about the skills and abilities of robots were assessed via 30 items (*robot skills and abilities*; e.g., “Robots are able to feel pain.”; 1 = “strongly disagree” to 5 = “strongly agree”; α = 0.90). Additionally, participants stated in how many years they expect robots to be more intelligent than humans, if at all. Furthermore the subscale *expectations for humanoid robots in daily life* (6 items; e.g., “Humanoid robots can make our lives easier.”; α = 0.77) of the Frankenstein Syndrome Questionnaire was employed (Nomura et al., 2012; 1 = “strongly disagree” to 5 = “strongly agree”).

#### Negative Expectancies

To assess participants’ negative expectancies regarding social robots, another subscale (*general anxiety toward humanoid robots*) of the Frankenstein Syndrome Questionnaire was used (13 items; e.g., “I don’t know why, but humanoid robots scare me.”; α = 0.89). Furthermore, 12 self-developed items asking about common situations from science fiction were employed (*negative expectancies regarding robots*, e.g., “Robots will try to free themselves from humans.”; 1 = “strongly disagree” to 5 = “strongly agree”; α = 0.86).

#### Technical Background

The two scales *locus of control when using technology* (KUT; Beier, 1999; 8 items; e.g., “I feel so helpless regarding technical devices that I rather keep my hands off of them.”; 1 = “strongly disagree” to 5 = “strongly agree”; α = 0.89) and t*echnical affinity as handling of and attitude toward electronic devices* (TA-EG; Karrer et al., 2009; 19 items; e.g., “I enjoy trying an electronic device.”; 1 = “does not apply at all” to 5 = “applies completely”; α = 0.86) were used to consider people’s technical background.

#### Demographical Background

Participants stated their age, sex, educational level, and current employment or training status.

### Results

Of the 433 participants, 82 (18.9%) people had previous contact with an industrial robot before, 265 (61.2%) with a vacuum cleaner or lawn mower robot, 46 (10,6%) with a social robot, and 32 (7.4%) with some other kind of robot. Most participants (77,1%) have seen at least one report or something similar about industrial robots before, 199 (46%) about vacuum cleaner or lawn mower robots, 225 (52%) about social robots, and 69 (15.9%) about some other kind of robots. On average, participants stated to be familiar with 7.06 (*SD* = 4.05) of the 16 presented movies and series and to remember 9.27 (*SD* = 6.12) of the 25 fictional robots. The participants stated to neither very often nor very rarely watch science fiction movies or series with robots in important roles (*M* = 2.58, *SD* = 1.32) and they rate the relationship between humans and robots in those movies and series in general neither clearly negative nor positive (*M* = 3.09, *SD* = 0.69). Besides the 187 participants (43.2%) which do not expect robots to ever be more intelligent than humans, the others expect robots to be more intelligent in an average of 63.47 years (*SD* = 177.45). For an overview of the descriptive values of the main influencing and dependent variables see Table 2. The complete data set can be found in Supplementary Data Sheet S1.

**Table 2:** Descriptive statistics of the main influencing and dependent variables.

		*M*	*SD*	α
**Influencing variables**	Contact with robots (frequency)	1.67	1.54	–


	Reception of reports about robots (frequency)	1.76	1.04	–
	Knowledge of fictional robots	9.27	6.12	–
	Knowledge of negatively perceived fictional robots	3.33	2.63	–
	Knowledge of positively perceived fictional robots	4.27	2.44	–
	Locus of control when using technology (KUT)	3.51	0.84	0.89
	Technical affinity (TA-EG)	3.33	0.59	0.86
**Dependent variables**	Robot skills and abilities (RSA)	2.67	0.55	0.90


	RSA: Human-like Robot Skills	1.56	0.61	0.90
	RSA: Machine-like Robot Skills	3.56	0.72	0.88
	Contact intentions	2.89	1.18	0.90
	Interaction expectations	3.25	0.92	0.92
	FSQ: General anxiety toward humanoid robots	2.92	0.84	0.89
	FSQ: Expectations for humanoid robots in daily life	2.86	0.79	0.77
	Negative expectancies regarding robots	2.62	0.74	0.86

**Figure 1:**
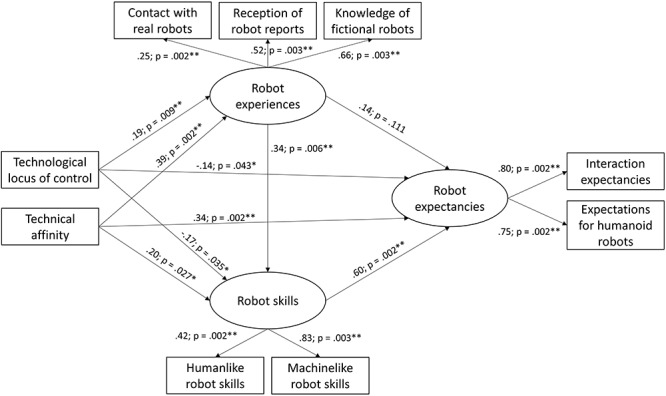
Structural equation model for robot expectancies. ^∗∗∗^*p* < 0.001, ^∗∗^*p* < 0.01, ^∗^*p* < 0.05.

To test the hypotheses 1 and 3a, a structural equation model (SEM) was built with the latent variables *robot experiences*, *robot skills*, and *robot expectancies* and the manifest variables *locus of control when using technology* and *technical affinity* (Figure 1). The SEM analysis was computed using IBM SPSS AMOS 25.0 for Windows (IBM SPSS statistics, released 2017).

The latent variable robot experiences consists of the three manifest variables *contact with real robots* (mean of the contact frequencies with the robot types people had interacted with before), *reception of robot reports* (mean of the report reception frequencies of the robot types people saw a report on before), and *knowledge of fictional robots* (number of fictional robot characters people recall). Robot skills compromises the two manifest variables *human-like robot skills* and *machine-like robot skills* (subscales of the robot skills and abilities scales, obtained via factor analysis). Robot experiences is represented by the two manifest variables *interaction expectancies* and *expectations for humanoid robots in daily life* (subscale of the FSQ). There were no missing data and the variables were tested for multivariate normality and multicollinearity. For the evaluation of the model fit, standard criteria were applied (Hu and Bentler, 1995, 1999). The root mean square error of approximation (RMSEA; values below 0.08 indicate an acceptable fit), comparative fit indices (CFI/TLI; values above 0.90 indicate a good fit), and the standardized root mean square residual (SRMR; values below 0.08 indicate a good fit with the data). For the current model, the RMSEA was 0.06, CFI was 0.96, TLI was 0.93, and the SRMR was 0.03, indicating a good model fit.

The standardized model results for the latent dimensions can be found in Table 3. There was a significant direct effect of robot experiences on robot skills (β = 0.36, *SE* = 0.11, *p* = 0.006), but not on robot expectancies (β = 0.14, *SE* = 0.09, *p* = 0.111). However, a significant direct effect of robot skills on robot expectancies was found (β = 0.60, *SE* = 0.08, *p* = 0.002) and there was a significant indirect effect of robot experiences on robot expectations via robot skills (β = 0.20, *SE* = 0.08, *p* = 0.003). Furthermore, technical affinity had a significant direct effect on robot experiences (β = 0.39, *SE* = 0.07, *p* = 0.002), robot skills (β = 0.20, *SE* = 0.09, *p* = 0.027), and robot expectancies (β = 0.34, *SE* = 0.07, *p* = 0.002). Locus of control had a significant direct effect on robot experiences (β = 0.19, *SE* = 0.08, *p* = 0.009), robot skills (β = -0.17, *SE* = 0.08, *p* = 0.035), and robot expectancies (β = -0.14, *SE* = 0.06, *p* = 0.043) as well. To sum up, participants’ experiences with robots, which is mainly influenced by people’s knowledge of fictional robots and their reception of reports about real robots (see Table 3), significantly enhanced their assessment of the skills and abilities of robots. This, in turn, significantly enhanced their general expectations of robots being part of their lives and the society in general (supporting hypothesis 1). In addition, participants’ technical affinity leads to stronger expectations regarding robots’ skills and regarding the role of robots, while their locus of control using technology reduces these expectations (supporting hypothesis 3a).

**Table 3:** Coefficients of the manifest variables’ loadings on the latent dimensions.

Latent dimension	Manifest variables	β	*SE*	*p*
Robot experiences	Contact with real robots	0.25	0.06	0.002^∗∗^
	Reception of robot reports	0.52	0.06	0.003^∗∗^
	Knowledge of fictional robots	0.66	0.06	0.003^∗∗^
Robot skills	Human-like robot skills	0.42	0.05	0.002^∗∗^
	Machine-like robot skills	0.83	0.07	0.003^∗∗^
Robot expectancies	Interaction expectancies	0.80	0.04	0.002^∗∗^
	Expectations for humanoid robots	0.75	0.04	0.002^∗∗^

^∗∗∗^*p < 0.001, ^∗∗^p < 0.01, ^∗^p < 0.05*.

Likewise, to test the hypotheses 2 and 3b, another SEM was built with the latent variables *robot experiences*, *robot skills*, and *robot fears* and the manifest variables *locus of control when using technology* and *technical affinity*. The latent variables robot experiences and robot skills were formed as described before, besides that the variable *knowledge of fictional robots* was separated into the two variables *knowledge of negatively perceived fictional robots* and *knowledge of positively perceived fictional robots* (obtained via factor analysis to extract and separate the negatively and positively perceived fictional robots and to identify and remove the fictional robots with ambiguous evaluations; eight robots loaded with a factor representing positively perceived robots: R2-D2, C-3PO, BB-8, Number 5, Bumblebee, Wall-E, Data, and David; nine robots loaded with a factor representing negatively perceived robots: Megatron, the Machines, the other robots of the type NS-5, the Cylons, the military robots S-A-I-N-T, Ultron, T-100, HAL 9000, and T-800; five robots were extracted since they loaded with factors which only represented the robots of one movie and had ambiguous evaluations: Rachael, Officer K, and Roy Batty from Blade Runner as well as Maeve and Dolores from Westworld; three robots were removed because they did not load with any factor sufficiently). Robot fears consisted of the two manifest variables *negative expectancies regarding robots* and *general anxiety toward humanoid robots* (subscale of the FSQ). For this model, the RMSEA was 0.10, CFI was 0.86, TLI was 0.78, and the SRMR was 0.09, indicating no acceptable model fit. Thus, this model needed to be rejected.

To still examine what could possibly have an influence on people’s fears regarding robots, two multiple hierarchical regression analyses were conducted. For both analyses the predictor variables knowledge of negatively perceived fictional robots (NegRob) knowledge of positively perceived fictional robots (PosRob) reception of robot reports, and contact with real robots were entered in Step 1, while technical affinity and locus of control using technology were added in Step 2. For the first regression analysis with negative expectancies regarding robots as dependent variable, the predictor variables explain 5% of the variance in the first step [*R*^2^ = 0.05, *F*(4,428) = 5.59, *p* < 0.001] and 5.6% in the second step [Δ*R*^2^ = 0.01, Δ*F*(2,426) = 1.53, *p* = 0.219]. Only knowledge of negatively perceived fictional robots emerged as significant predictor (β = 0.28, *p* < 0.001), suggesting that the more negatively perceived fictional robots people know of, the more they fear robots to become a threat to humanity (partly supporting hypothesis 2a). In addition, contact with real robots had a marginally significant influence (β = -0.09, *p* = 0.064), indicating a reducing effect on people’s negative expectancies (partly supporting hypothesis 2a; hypothesis 3b is not supported).

Looking at the second regression analysis with general anxiety toward humanoid robots as dependent variable, the predictor variables in the first step explain 5.2% of the variance [*R*^2^ = 0.05, *F*(4,428) = 5.84, *p* < 0.001] and in the second step 17.1% [Δ*R*^2^ = 0.12, Δ*F*(2,426) = 30.54, *p* < 0.001]. Only technical affinity (β = -0.32, *p* < 0.001) emerged as significant predictor, suggesting that the more technophile people are, the less general fears about robots becoming part of the society do they have (partly supporting hypothesis 3b). Furthermore, the knowledge of positively perceived fictional robots (β = -0.11, *p* = 0.096), the reception of reports about real robots (β = -0.09, *p* = 0.069), and locus of control when using technology (β = -0.09, *p* = 0.094) had a marginally significant influence. This indicates that the more positively perceived fictional robots people know, the more frequently they watched reports about real robots and the higher their technological locus of control is, the less general fears do they have (partly supporting hypothesis 2b and 3b). The results of both regression analyses are presented in Table 4.

**Table 4:** Coefficients for the hierarchical regression analyses with (a) negative expectancies regarding robots and (b) general anxiety toward humanoid robots as criterion.

	Negative expectancies					General anxiety				
	Δ*R*^2^	*b*	*SE B*	β	*p*	Δ*R^2^*	*b*	*SE B*	β	*p*
**Step 1**	0.05					0.05				
Constant		2.53	0.09		<0.001***		3.35	0.10		<0.001***
NegRob		0.08	0.02	0.27	<0.001***		0.01	0.02	0.03	0.685
PosRob		-0.03	0.02	-0.09	0.218		-0.06	0.03	-0.16	0.025*
Reports		0.02	0.04	0.03	0.600		-0.12	0.04	-0.14	0.005**
Contact		-0.05	0.02	-0.10	0.034*		-0.02	0.03	-0.03	0.553
**Step 2**	0.01					0.12				
Constant		2.85	0.21		<0.001***		4.91	0.22		<0.001***
NegRob		0.08	0.02	0.28	<0.001***		0.03	0.02	0.08	0.230
PosRob		-0.02	0.02	-0.08	0.292		-0.04	0.02	-0.11	0.096^†^
Reports		0.03	0.04	0.04	0.442		-0.07	0.04	-0.09	0.069^†^
Contact		-0.04	0.02	-0.09	0.064^†^		0.01	0.03	0.03	0.586
TA-EG		-0.11	0.07	-0.09	0.140		-0.45	0.08	-0.32	<0.001***
KUT		-0.00	0.05	-0.00	0.949		-0.09	0.05	-0.09	0.094^†^

^∗∗∗^*p < 0.001, ^∗∗^p < 0.01, ^∗^p < 0.05, ^†^p < 0.1. NegRob = knowledge of negatively perceived fictional robots, PosRob = knowledge of positively perceived fictional robots, TA-EG = technical affinity, KUT = locus of control when using technology*.

## Discussion

Even though the general interest in robots is increasing and consequently more and more robots are becoming available, social robots are still a rather new and unfamiliar technology to the majority of people (Van Oers and Wesselmann, 2016). In addition, it is challenging for people to assign a clear category to social robots (Kahn et al., 2011) since they often look and behave like a human or at least like some kind of living being, even though they are non-living electronic devices. These circumstances probably elicit a lot of uncertainty which people usually attempt to reduce in interpersonal situations by gathering more information (Berger and Calabrese, 1975) and trying to form an impression (Asch, 1946), often based on social categories (Andersen and Klatzky, 1987). Consequently, people probably also attempt to form expectations of social robots by considering all available sources of information.

By means of the conducted interviews, people’s expectations regarding social robots in their personal daily lives and regarding social robots having own emotions and intentions were examined more deeply. Moreover, these expectations were systematically compared to their preferences, which has not been examined this way before. Mostly, people expect and would like social robots to make their lives easier by helping with household or work-related tasks, but not with social activities, to have human skills and abilities, but to be clearly distinguishable from humans, and to have no own emotions or intentions. However, people do mention more emotional and interpersonal abilities in contrast to technological abilities when talking about preferences and in particular wish for empathetic social robots, which they do not actually expect. Thus, when talking about expectancies, people seem to focus more on technological aspects, while their preferences represent rather emotional and social aspects. It is also interesting that empathy, an emotion which benefits and is directed at humans, is something people wish for but not expect. Meanwhile, other emotions which are more related to the robot itself and do not benefit any person, like anger or sadness, are neither expected nor desired.

An online study was conducted to further examine how and on what bases people form their expectations regarding social robots and specifically what influence science fiction movies or series have on these expectations. The results of the online study indicate that people’s experiences with robots, meaning their actual contact with different kinds of real robots, but also their reception of media coverage on real robots and their knowledge of fictional robot characters, have an influence on people’s assessment of robots’ skills and abilities. Further, this assessment influences whether people in general expect to interact with robots in their daily life and to what extent they expect robots to become part of the society. Especially people’s media reception played an important role in the formation of their expectancies of social robots, which now systematically confirms what was indicated by previous research (Khan, 1998; Bruckenberger et al., 2013; Sandoval et al., 2014). The results of the study show that most people did not have much contact with a real robot before, if at all. Hence, it seems natural that people mainly base their expectations on what they take in from mass media. These appear to be primarily science fiction movies and series, but also reports and similar formats on real robots. Also in line with previous assumptions, people appear to be biased in their expectancies regarding robot skills and abilities (Bruckenberger et al., 2013; Sandoval et al., 2014), probably because robots are usually portrayed with unrealistically high developed skills in the media, especially in science fiction formats. Since people have the strong need to reduce uncertainty and to increase predictability (Berger and Calabrese, 1975), a possibly unreliable or biased source appears to be favorized over having no source and consequently being completely unknowing and unprepared. Moreover, technophile people, who are by definition enthusiastic about new technologies, appear to be especially inclined to have high expectations regarding robots’ skills and abilities and whether robots will be part of their lives and the society in general. These people are probably so fascinated by social robots as a special form of new technology that they are not so much interested in what they can realistically do, but more in what skills would be exciting for them to have. This could explain the rather high expectations of technophile people regarding social robots. Locus of control when using technology, which probably for the most part reflects people’s actual competence regarding the usage of technologies (Beier, 1999), has the opposite effect. People with a high technological locus of control are probably more able to make a realistic assessment of what social robots are and will be capable of due to their general understanding of technological devices.

Regarding people’s negative expectancies and fears of robots, the proposed model failed to explain the possible structure of influences. It seems that these fears are not that easily explained and may have a variety of sources and triggers. However, we were still able to discover a few connections. First of all, negative expectancies (reflecting common science fiction scenarios about robots breaking free and becoming a threat to humans) were mainly predicted by people’s knowledge of negatively perceived fictional robot characters. Consequently, it appears that the more negatively perceived fictional robots people remember, the more do they expect robots to become dangerous for humans, which reflects the common theme of those science fiction formats with negatively portrayed robot characters. This also matches the results of Ray et al. (2008), where respondents mentioned fears resembling common science fiction scenarios. People who had contact with real robots before, however, appear to be less worried about potential dangers coming from robots. Contact seems to reduce negative prejudices regarding robot interaction partners, which was already found to be the case between human interaction partners (Binder et al., 2009). Contact is considered to be one of the most effective strategies to reduce intergroup bias and conflicts and to improve intergroup relations (Dovidio et al., 2003), which is also known as contact hypothesis (Allport, 1954). However, more likely people’s illusion about the skills and abilities of social robots were more grounded when they actually met a real robot and noticed that they are far away from the depiction of robots in movies and series. Moreover, one of the axioms of the uncertainty reduction theory states that decreases in uncertainty level produce increases in liking (and the other way around; Berger and Calabrese, 1975). Thus, contact with social robots probably reduces uncertainty which results in less negative feelings toward them.

With regard to people’s general anxiety toward humanoid robots, primarily technical affinity was found to have a reducing effect. Technical affinity describes a very positive or even enthusiastic attitude toward new technologies (Karrer et al., 2009), thus it is not surprising that technophile people have less fears regarding these technologies, in this case regarding social robots. People’s knowledge of positively perceived fictional robots also had a reducing effect on their general anxiety. Knowing many “good” robots from science fiction probably results in a generally rather positive picture of robots. Additionally, people’s reception of robot reports and their technological locus of control also came along with less general anxiety, which is probably because here again people were more informed what technological devices like social robots are capable of and thus less afraid of them.

### Limitations and Future Research

One limitation of both studies is that participants were exclusively recruited through Internet-based technologies and were predominantly young and highly educated, which could affect the generalizability of the results. Moreover, only people with a similar European cultural background were examined. Since people’s cultural background can impact their attitudes and expectations as well as their actual behavior during an interaction with a robot (e.g., Kaplan, 2004; Bartneck et al., 2006; Li et al., 2010), future studies should also consider this as another pivotal influence. Furthermore, due to the cross-sectional nature of the studies no conclusions regarding causal relationships can be derived. Also, there may be further information sources people use to form expectations of social robots than the ones that were assessed in the current study, which would be interesting to explore in future studies. For the online study, the sequence of the questionnaires was not randomized, thus, questions regarding participants’ experiences especially with the fictional robots might have affected their answers regarding their general and negative expectancies. Since all results are based on self-evaluation and thus mainly reflect what people think they expect, it would be of interest to also examine what kinds of implicit expectations and maybe also prejudices people have regarding robots and how these influence people’s behavior for example during an actual interaction with a robot. In this study contact seemed to reduce negative expectancies, however, not many people actually had contact with a robot before. Therefore, the influence of contact on people’s expectations should be examined further, especially since research showed that different effects are possible (Wullenkord et al., 2016). For the current study, only the overall influences of the different parameters were analyzed. Controlling for one variable, e.g., knowledge of fictional robots, could deliver further and more specific insights. Also, it would be interesting to find out more about how people form their expectations when they are first meeting a social robot and how they react if their initial expectations are violated by conducting experimental lab studies with an actual robot.

### Conclusion

In general, people’s expectations also resemble their preferences regarding social robots with the exception of empathy, which is desired but not expected. Since it is difficult to assign a clear, existing category to social robots, it stands to reason that people attempt to form a variety of expectations by using all information sources that are available to them to reduce their uncertainty. Since most people have never interacted with a social robot before, other sources of information like mass media are used to form expectancies regarding the skills of robots and, based on that, regarding the role of robots in their personal life and in society in general. However, the reception of “bad” fictional robots appears to elicit fears which reflect common scenarios from science fiction, which can possibly be reduced through actual contact with real robots. These findings should be considered when developing and designing social robots, since people’s expectancies will influence their attitude toward and evaluation of social robots. It should be kept in mind that especially people’s assessment of robots’ skills and abilities play a major role, whereby people’s fears may be reduced when they recognize that robots are not as far developed as the robots portrayed in science fiction. Consequently, further work should also be invested into education and a more realistic representation of social robots in the media so that the public is also able to develop more realistic expectations. According to Weiss et al. (2011b, p.121), “in public presentations of robots, uncertainties and unsolved technological problems in robotics usually remain black boxed.” Moreover, media reports about real robots are less ubiquitous than science fiction formats and therefore also consumed less (Bruckenberger et al., 2013). Hence, there should be more accessible media coverage on real social robots showing what they are presently able to do, but also showing current technological as well as developmental boundaries and problems.

## Ethics Statement

This study was carried out in accordance with the recommendations of the ‘ethics committee of the Division of Computer Science and Applied Cognitive Sciences at the Faculty of Engineering of the University of Duisburg-Essen’ with written informed consent from all subjects. All subjects gave written informed consent in accordance with the Declaration of Helsinki. The protocol was approved by the ‘ethics committee of the Division of Computer Science and Applied Cognitive Sciences at the Faculty of Engineering of the University of Duisburg-Essen’.

## Author Contributions

AH and NK: conceptualization, methodology, resources, validation, writing, review, and editing. AH: data curation, formal analysis, investigation, project administration, software, visualization, writing, and original draft. NK: supervision.

## Conflict of Interest Statement

The authors declare that the research was conducted in the absence of any commercial or financial relationships that could be construed as a potential conflict of interest.
