# Supplementary material for: Great Expectations? Relation of Previous Experiences With Social Robots in Real Life or in the Media and Expectancies Based on Qualitative and Quantitative Assessment
Source: Front Psychol. 2019 Apr 30;10:939. doi: 10.3389/fpsyg.2019.00939 (PMC6503083; doi:10.3389/fpsyg.2019.00939)
Supplement: Supplementary file 1 [file Table_1.DOCX]

**Interview questions: Robot expectancies and preferences**

1. Warming up:
   1. In general, what is your attitude towards new technologies? (Rather open/enthusiastic or skeptical/reserved?)
   2. Did you have contact with a robot before? Where and when?
2. Please describe to me a typical day in your life. Start with your morning and describe all of your activities until you go to bed again in the evening. Write down one keyword for each activity. […] Please take another look at your activities.
   1. During which activities do you expect an interaction with a robot in the future?
   2. During which not?

By robots I do not mean industrial robots, but robots which are able to interact socially with humans. From now on we will call this kind of robots “social robots”.

1. Imagine you would live in the year of 2068 (50 years in the future).
   1. In which areas do you expect to encounter social robots?
   2. Which kinds of robots do you expect? Domestic robots, office robots, public service robots, health care robots or something completely different?
   3. Which skills do you think social robots will have? Which not?
   4. How do you think social robots will behave? (Rather like machines or like living beings?)
   5. Will social robots have emotions?
   6. Will social robots have own intentions, meaning an own will?
2. Imagine you could decide freely how a social robot behaves in an interaction with you and what it is capable of.
   1. Which skills do you wish for the robot to have? Which should it not have?
   2. How do you wish should the social robot behave? (Rather like a machine or like a living being?)
   3. Should the social robot show and have emotion? Why (not)?
   4. Should the social robot have own intentions, meaning an own will? Why (not)?
3. Imagine the following situation: You are coming home from school/work. Arriving in your apartment, your social robot welcomes you. You instruct your robot to clean up the apartment. Instead of following your instructions immediately, it stands still and says: “I would much rather hear how your day was and what you experienced. In this apartment I am so cut off. Please tell me about the world outside! I was looking forward to this all day.”
   1. How do you like this idea?
   2. What do you feel imagining this?
   3. What thoughts are going through your mind?
4. Summing up:
   1. Where do you see the greatest advantage/benefit of social robots?
   2. Where do you see the greatest disadvantage/danger of social robots?
